# Inhibiting trained immunity in Duchenne muscular dystrophy enhances muscle regeneration and reduces fat deposition

**DOI:** 10.21203/rs.3.rs-10008020/v1

**Published:** 2026-08-14

**Authors:** Lorenza Esposito, Andrea Bracaglia, Anna Picca, Giorgia Cavioli, Veronica Ruggieri, Carmine Nicoletti, Luca Madaro, Carles Sanchez Riera, Dario Coletti, Anna Ranzenigo, Giulia Maria Davighi, William Wang, Judit Morla-Folch, Nicola Tumino, Paola Vacca, Emanuele Marzetti, Marina Bouche, Abram JP Teunissen, Biliana Lozanoska-Ochser

**Affiliations:** Sapienza University of Rome: Universita degli Studi di Roma La Sapienza; Sapienza University of Rome: Universita degli Studi di Roma La Sapienza; Fondazione Policlinico Universitario Agostino Gemelli IRCCS; Sapienza University of Rome: Universita degli Studi di Roma La Sapienza; Sapienza University of Rome: Universita degli Studi di Roma La Sapienza; Sapienza University of Rome: Universita degli Studi di Roma La Sapienza; Sapienza University of Rome: Universita degli Studi di Roma La Sapienza; Sapienza University of Rome: Universita degli Studi di Roma La Sapienza; Sapienza University of Rome: Universita degli Studi di Roma La Sapienza; Icahn School of Medicine at Mount Sinai; Icahn School of Medicine at Mount Sinai; Icahn School of Medicine at Mount Sinai; Icahn School of Medicine at Mount Sinai; Bambino Gesu Pediatric Hospital: Ospedale Pediatrico Bambino Gesu; Bambino Gesu Pediatric Hospital: Ospedale Pediatrico Bambino Gesu; Fondazione Policlinico Universitario Agostino Gemelli IRCCS; Sapienza University of Rome: Universita degli Studi di Roma La Sapienza; Icahn School of Medicine at Mount Sinai; UniCamillus: Saint Camillus International University of Health and Medical Sciences

**Keywords:** Trained immunity, inflammatory monocytes, DMD, mTORi nanobiologics

## Abstract

**Background:**

Trained immunity (TI) refers to a hyperresponsive functional state of innate immune cells, such as inflammatory monocytes, induced in response to a variety of stimuli including signals released from damaged tissue, and characterised by increased cytokine production. Inflammatory monocytes play a critical role in Duchenne muscular dystrophy (DMD) by driving chronic muscle inflammation and disease progression. In this study we examined the role of trained immunity in dystrophic muscle pathology by specifically targeting this process in monocytes.

**Methods:**

We used the *mdx* mouse model of DMD to investigate TI induction in splenic and bone marrow monocytes isolated during the peak of muscle injury and inflammation. Transcriptional changes in monocytes were examined by performing RNA-Seq, while cytokine production following stimulation with LPS was examined by multiplex ELISA. Mice were treated for two weeks with twice weekly i.v. injections of apolipoprotein A1–based, rapamycin-loaded nanobiologics (mTORi-NB) to specifically target TI in monocytes within the bone marrow and the spleen through the inhibition of the mTOR pathway which plays a central role in TI induction. Inhibition of TI in monocytes was assessed *in vitro* by measuring cytokine production following stimulation with LPS. Satellite cells and FAPs were sorted from limb muscles and cultured *in vitro* to assess the effect of the treatment on their proliferation and differentiation properties.

**Results:**

Dystrophic muscle injury was associated with transcriptional changes in *mdx* monocytes linked to chromatin and metabolic remodelling as well as with hyperproduction of proinflammatory cytokines, consistent with TI induction. Treatment of *mdx* mice with mTORi-NB resulted in improved skeletal muscle regeneration and reduced fat deposition. *In vitro* experiments confirmed the enhanced myogenic properties in satellite cells and reduced adipogenesis in FAPs mediated by the inhibition of TI in monocytes.

**Conclusions:**

Dystrophic muscle injury induces TI in both bone marrow and splenic monocytes characterised by hyperproduction of proinflammatory cytokines. Targeting TI specifically in monocytes through mTOR inhibition enhances muscle regeneration and reduces intramuscular fat deposition. Overall, our findings suggest that inhibition of TI represents a promising therapeutic strategy to slow DMD progression.

## Background

Duchenne muscular dystrophy (DMD) is a severe X-linked muscular disorder caused by mutations in the dystrophin gene, leading to the absence of functional dystrophin and progressive muscle degeneration (1). Dystrophin is a membrane-associated cytoskeletal protein, essential for maintaining sarcolemma integrity; its loss renders myofibers highly unstable and prone to contraction-induced membrane damage. Repeated cycles of muscle injury promote the release of damage-associated molecular patterns (DAMPs), which triggers chronic activation of innate immune pathways and a persistent inflammatory milieu that exacerbates disease progression (2). Numerous studies have identified inflammatory monocytes as major contributors to dystrophic muscle pathology, with their sustained recruitment and chronic activation driving progressive myofiber degeneration and fibrosis (3-5). In healthy skeletal muscle, tissue repair is coordinated through finely tuned interactions between inflammatory monocyte-derived macrophages (Mo-MP), muscle stem cells (satellite cells; MuSCs), and fibro-adipogenic progenitors (FAPs). Following acute injury, inflammatory monocytes are transiently recruited to damaged muscle tissue, where they differentiate into pro-inflammatory macrophages that clear necrotic myofiber debris and stimulate MuSCs activation and proliferation through the production of pro-inflammatory cytokines including TNF-α, IL-1β, and IL-6 (5, 6). Subsequently, these macrophages undergo a tightly regulated transition toward pro-regenerative phenotypes characterized by the secretion of anti-inflammatory mediators such as IL-10, and promote SC differentiation, myoblasts fusion, and the formation of new myofibers (6). Likewise, Mo-MP tightly regulate the activation, expansion and subsequent clearance of FAPs, preventing excessive fibrotic and fat tissue deposition (7). In DMD, however, recurrent cycles of myofiber degeneration drive persistent recruitment and activation of inflammatory monocytes, resulting in impaired SC regenerative capacity, and sustained FAPs activation and survival. Emerging evidence further suggests that inflammatory monocytes in dystrophic muscle may acquire features of trained immunity (TI). TI is a type of non-specific immune memory induced in innate immune cells such as monocytes that results in enhanced cytokine responses after secondary stimulation (8, 9). The TI phenotype depends on activation of the mammalian target of rapamycin (mTOR) pathway and relies on epigenetic and metabolic reprogramming in myeloid progenitors in the bone marrow (BM) and spleen, giving rise to hyper-responsive monocytes (10). In addition to microbial stimuli, endogenous signals such as cytokines (i.e., IL-1β, GM-CSF and IFN-γ) and DAMPs (11) have also been linked to TI induction in monocytes.

While protective against infections (12, 13), persistent activation of TI has been associated with worsening of chronic inflammatory disorders such as atherosclerosis, cardiovascular disease, and autoimmunity (14-17). Furthermore, in a recent study, Bhattarai *et al.* demonstrated that BM-derived macrophages from the *mdx* mouse model of DMD display features consistent with TI and suggested that increased cytokine production by trained inflammatory monocytes may exacerbate chronic inflammation and contribute to disease progression (18). However, the extent to which TI drives chronic muscle injury in DMD, and whether it can be therapeutically modulated, remains largely unexplored.

We recently developed apolipoprotein A1 (apoA-I)-based nanobiologics that preferentially target myeloid cells in hematopoietic organs, enabling cell-specific delivery of immunomodulatory cargo while minimizing systemic toxicity (19-21). When loaded with rapamycin these nanobiologics inhibit the mTOR pathway (mTORi-NB), which plays a central role in TI (10). Of note, the clinical translatability of these nanobiologics has already been demonstrated by scaling production, for example apoA-I has been produced (in sufficient amounts) for safe application in humans and toxicology studies in nonhuman primate models (19, 21-23).

The ability of mTORi-NBs to selectively inhibit TI in inflammatory monocytes was recently demonstrated in a mouse model of heart transplantation (21, 23, 24), where treatment prolonged allograft survival, and atherosclerosis, where it significantly reduced chronic inflammation (24). Here, we extend those findings by showing that inhibition of TI in inflammatory monocytes with mTORi-NB in the *mdx* mouse model of DMD reduces dystrophic muscle pathology by ameliorating muscle fiber regeneration and reducing intramuscular fat deposition.

## Methods

### Mice

C57BL/6J and C57BL/10ScSn-Dmdmdx mice were purchased from the Jackson laboratory (Bar Harbor, ME, USA). Only male mice were used. The mice were housed in the histology department-accredited animal facility at the University of Sapienza. Mice were randomly allocated to control and intervention groups. All the procedures were approved by the Italian Ministry for Health and were conducted according to EU regulations and the Italian Law on Animal Research. The study is compliant with all relevant ethical regulations regarding animal research as well as the principles of the 3Rs (Replacement, Reduction and Refinement).

### Isolation of monocyte from spleen and bone marrow

Monocytes were isolated from the spleen and BM of wild-type (WT) uninjured mice and *mdx* mice at different ages (from 2 to 8 weeks of age) and experimental conditions (mTORi-NBs and empty-NBs treatment). Splenocytes were obtained by grinding the spleen between the rough edges of 2 microscopy slides (Thermo Fisher Scientific), while BM cells were flushed from femurs and tibias by centrifuging at 10.000 rpm for 30 sec. Following the lysis of RBCs with PBS 10x (PanReac AppliChem) and ddH2O, cells were resuspended in RPMI-1640 (Sigma Aldrich) 10% FBS (Life Technologies). Cell suspensions were obtained by passing through a 40 μm cell strainer (BD Falcon). BM and splenic monocytes were isolated using the miltenyi negative selection kit (Miltenyi Biotec) according to the manufacture’s protocols or the pre-plating method (25) . Briefly, BM cells were plated at a density of 3 x 10^5^ cells per well in a 96 well (BD Falcon) and incubated for 3 hours at 37 °C, 5% CO_2_ in RPMI 10% FBS. After preplating, non-adherent cells were removed with PBS with Ca^2+^/Mg^2+^ (Sigma Aldrich) and the adhered monocytes were cultured in RPMI-1640 medium supplemented with 10% fetal bovine serum (FBS), 1% penicillin-streptomycin (Euroclone), 1% glutamine, sodium pyruvate (Sigma Aldrich) and M-CSF (R&D systems) at 37 °C , 5% CO_2_ for 7 days (the growth medium was changed on day 3 of culture), to allow differentiation into monocyte-derived macrophages (Mo-MP). After 7 days, Mo-MP were stimulated with lipopolysaccharide (LPS, 100 ng/ml) for 4 or 24 hours according to the experimental design. Splenocytes were cultured at a density of 1 x 10^6^ cells per well in a 48-well (BD Falcon) and subjected to two sequential preplating steps (1 hour each at 37 °C, 5% CO_2_), as described above.

Control wells were maintained in parallel under identical conditions without LPS stimulation. Following stimulation, cells and supernatants were collected for downstream analyses, including gene expression profiling and cytokine quantification.

### RNA seq

Total RNA from splenic Mo-MP was extracted using Qiagen RNeasy mini kits following the manufacturer’s protocol. RNA was sent to The Institute of Applied Genomics (IGA) of Udine (IT) (https://appliedgenomics.org/it/) for sequencing. The libraries for sequencing were prepared using the Illumina kit and sequenced with Illumina NovaSeq X Plus (paired end 150pb). For each biological condition three independent experiments were carried out for the isolation of RNA. Base calling and demultiplexing were carried out with BCL Converter v4.3.13. Low quality bases and adapters were then removed with Trim Galore (v0.6.10). Subsequently, reads were aligned to mouse transcriptome GRCm39 (mm39) using STAR with default parameters and counts tables were generated using featureCounts program. Differential expression of genes was conducted using the R package DESeq2 (v1.50.2). For downstream bioinformatic analysis and Gene Ontology only differentially expressed genes with adjusted p value <0.05 and log2FC >0.5 or <−0.5 were considered.

### Multiplex immunoassay

A panel of 23 inflammatory mediators was measured in the supernatant of cultured splenic Mo-MP following or not LPS stimulation using the commercially available Bio-Plex Pro Mouse Cytokine 23-plex immunoassay kit (#M60009RDPD), following the manufacturer’s protocol. The panel included cytokines such as IL-1a, IL-1β, IL-2, IL-3, IL-4, IL-5, IL-6, IL-9, IL-10, IL-12(p40), IL-12(p70), IL-13, IL-17A, TNF-α, IFN-γ, G-CSF, GM-CSF and chemokines such as MCP-1, MIP-1a, MIP-1b, Eotaxin, RANTES, KC. Data acquisition performed on a Bio-Plex^®^ System with Luminex xMap Technology (Bio-Rad). Defaults settings of the instrument were used and concentrations were calculated using Bio-Plex Manager Software (Bio-Rad). Before data export, optimization of standard curves across all analytes was performed and outliers were removed.

### ELISA assay

Cytokine levels in cell culture supernatants were quantified using enzyme-linked immunosorbent assays (ELISA). After LPS stimulation (4 hours), supernatants from monocyte-derived macrophage cultures were collected and stored at −80 °C until analysis. Concentrations of cytokines, including TNF-α and IL-6, were measured using commercially available mouse ELISA kits (R&D systems), according to the manufacturer’s instructions. Absorbance was read at 450 nm with a reference wavelength of 570 nm using a microplate reader. (Digital and analog systems s.r.l.) Cytokine concentrations were calculated from standard curves generated using recombinant standards provided in the kits and expressed as pg/mL.

### Treatment with mTORi-NB

mTORi-NB were made as reported (21) and administered at 5 mg/kg (in 0.1 ml PBS solution) via tail vein injection using a 28-gauge needle. C57BL10 ScSn-Dmdmdx/J mice were treated with the nanoparticles by twice weekly tail vein injections starting at 4-6 weeks of age for 2 weeks. Untreated control mice received empty NBs or PBS vehicle. At the end of the treatment tissues were harvested for FACS, histological and molecular analysis.

### Nanobiologics cellular uptake

DiO-labeled nanobiologics (DiO-NBs) were administered to C57BL10 ScSn-Dmdmdx/J mice at a dose of 5 mg/kg (in 0.1 ml PBS solution) via tail vein injection using a 28-gauge needle. 24 hours after administration, skeletal muscle, spleen and bone marrow were collected for flow cytometry analysis. Tissue processing and FACS procedures were performed as described below.

### Histological analysis

For immunofluorescence, tibialis anterior muscles were embedded in tissue freezing medium and snap-frozen in liquid nitrogen-cooled isopentane (Sigma Aldrich). 10 μm muscle cryosections were permeabilized with 100% acetone (Sigma Aldrich) for 1 min or PFA 4% (MilliporeSigma) for 10 minutes at RT followed by blocking for 1 h with a blocking solution containing 4% BSA (MilliporeSigma, A7030-100G) in PBS 1X. Primary antibodies were diluted in blocking solution, with incubation performed overnight at 4 °C. Antibody binding specificity was revealed using secondary antibodies coupled to Alexa Fluor 488, 594 or 555, or mouse monoclonal antibody from the Development Studies Hybridoma Bank (DSHB, Iowa, USA) for IgG (1:300). Incubation with secondary antibody was carried out at room temperature for 1 h.

The primary antibodies used for immunofluorescence included: anti-laminin (1:300, MilliporeSigma, L9393) and mouse monoclonal antibody from the Development Studies Hybridoma Bank (DSHB, Iowa, USA) for eMHC (1:20, DSHB, F1.652)

After immunostaining, sections were incubated with DAPI (Thermo Fisher Scientific, D1306) in PBS for 5 min for nuclear staining, washed in PBS, and mounted with glycerol (3:1 in PBS). Transverse sections were visualized on a Zeiss confocal microscope and images acquired using a Zeiss Imager A2. Muscle fibers cross-sectional area (CSA) analyses were performed using ImageJ software. All reported images are representative of all examined fields.

For Oil Red O (ORO) staining cryosections were fixed in 4% PFA for 1 h at room temperature, washed with H_2_Odd, and then in 60% isopropanol for 10 min at RT. Sections were dried and then stained with ORO solution in 60% isopropanol for 30 min at RT. After 3 x washes with tap water, slides were mounted in 10% glycerol solution. Images were acquired using a Zeiss Imager A2.

For Hematoxylin and Eosin staining, muscles cryosections were fixed in PFA 4% for 15 min at and then stained with Mayer’s Hematoxylin for 8 min followed by washing under running tap water for 10’. Eosin counterstain was applied for 1’ a RT. Following staining, slides were dehydrated by 75%, 90% and absolute alcohol cleared in Xylene and mounted with Eukitt (Sigma Aldrich, 03989). Images were acquired using a Zeiss Imager A2.

For sirius red staining, muscle cryosections were fixed for an 1 h at 56 °C in Bouin’s Solution (Sigma) and then stained in Picrosirius red solution (0.1%) (Sigma) for 1 h protected from light. After a brief washing step in acidified water 0.5% v/v, sections were fixed in 100% ethanol (Sigma) and a final dehydration step was performed in xylene 100%. Sections were mounted with EUKITT^®^. Images were acquired using a Zeiss Imager A2.

### TUNEL assay

Apoptosis was assessed using the In Situ Cell Death Detection Kit (Roche) according to the manufacturer’s instructions. Briefly, cultured monocytes were fixed with 4% PFA for 15 min at room temperature. Cells were then permeabilized with 0.1% Triton X-100 in 0.1% sodium citrate for 2 minutes on ice. Following permeabilization monocytes were incubated with TUNEL reaction mixture for 60 min at 37°C in a humidified chamber in the dark. Images were acquired using a Zeiss confocal microscope

### Quantitative Real-Time PCR

Total RNA was extracted from frozen muscle and spleen cells suspension using Tri-reagent (Zymo Research) and RNAeasy mini kits (Qiagen) according to the manufacturer’s instructions. RNA quantification was performed using a NanoDrop ONEc spectrophotometer (Thermo Scientific). First-strand cDNA was synthesized from the extracted RNA using a PrimeScript RT Reagent Kit with gDNA Eraser (Takara) following the manufacturer’s protocols. mRNA expression levels were determined by quantitative real-time PCR via QuantStudio 7 Flex (Applied Biosystems, Thermo Scientific) system using the ExcelTaq 2X Fast Q-PCR Master Mix (SYBR, ROX) (Smobio). Relative gene expression was calculated using the 2-DDCT method and normalized to the expression of the housekeeping gene actin beta (ActB).

**Table:** 

Primer list	
IL1β	FW: AGTGTGGATCCCAAGCA RV: CACTGTTGTTTCCCAGGA;
Pax7	FW: GTCCCAGTCTTACTGCCAC, RV: TGTGGACAGGCTCACGTTTT
MyoD	FW: CGCGCTCCAACTGCTCTGATGG RV: CTCGACACAGCCGCACTCTTCC
MyoG	FW: GTCCCAACCCAGGAGATCAT RV: GCTGTCCACGATGGACGTAA
ActB	FW: CACTGTCGAGTCGCGTCC RV: TCATCCATGGCGAACTGGTG

### Isolation and culture of satellite cells and FAPs

Satellite cells and FAPs were FACS-sorted from muscle as previously described (26). Briefly, tibialis anterior and gastrocnemius muscles were dissected with scissors and finely diced with a scalpel into 1-mm pieces. Processed muscles were then subjected to enzymatic digestion in PBS with 2 mg/mL Collagenase A (Roche), 2.4 units/mL Dispase I (Roche), 10 ng/mL DNase (Sigma Aldrich), 0.4 mM CaCl2 and 5 mM MgCl2 for 30 min at 37 °C. The cell suspension was filtered through a 100 μm and 40 μm nylon filter (BD Falcon) and incubated on ice with the following antibodies for 30 min: CD45 (1:50, eFluor 450, eBioscience), CD31 (1:50, eFluor 450, eBioscience), TER119 (1:50, eFluor 450, eBioscience), Sca1 (1:50,FITC, cloneD7, eBiosceince), Itga7 (1:500,AbLab).

Satellite cells were identified as TER119−/CD45−/CD31−/Itga7+/Sca1− cells; FAPs were isolated as TER-119−/CD45−/CD31−/Itga7−/SCA-1+ cells.

After isolation, satellite cells were counted, washed and resuspended in growth medium (GM) containing DMEM + Pyruvate (Gibco), 20% fetal bovine serum (FBS, Life Technologies), 10% horse serum (HS, Gibco), 1% penicillin/streptomycin (P/S,Euroclone) and 1% chicken embryo extract (CEE), and cultured in GM for 3 days, after which, cells were switched to differentiation medium (DM) containing DMEM +Pyruvate, 5% HS, 1% P/S for 24 hours and then stopped for IF analysis. To analyse the morphology of the myotubes, they were fixed in 4% PFA (MilliporeSigma) for 10 min at room temperature and then permeabilized with 0.1% Triton X-100 (Sigma Aldrich) for another 10 min. The myotubes were then blocked for 1 h with a solution containing 4% BSA (Millipore Sigma) in PBS 1X (PanReac Apllichem). The primary antibody (anti-Myosin; 1:20, DSHB) immunostaining was performed overnight at 4 °C, and antibody binding specificity was revealed using a secondary antibody coupled to Alexa Fluor 488 (goat anti-rabbit Alexa Fluor 488, Invitrogen). Nuclear staining was performed by incubating cells with DAPI in PBS for 15 min. Images were acquired using a Zeiss confocal microscope. The morphological analysis of myotubes size was conducted using ImageJ software. The mean value of these measurements was used for the subsequent statistical analysis. The reported figures are representative of all the examined fields.

After isolation, FAPs were counted, washed and resuspended in culture medium containing DMEM + Pyruvate (Gibco), 20% FBS (Life Technology) and 1% P/S (Euroclone) or culture medium plus conditioned medium from Mo-MP at different conditions, at a ratio of 1:2. Culture medium and CM were changed every 3 days. Cells were stopped 6 days after culture for IF analysis. FAPs were fixed in 4% PFA (Millipore Sigma) for 10 min at room temperature, followed by permeabilization with 0.1% Triton X-100 (Sigma Aldrich) for 15 min, and incubation in blocking solution 4% BSA (Millipore Sigma), 0.1% Triton X-100 and 10% goat-serum in PBS 1X for 1 h. The primary antibody (anti-perilipin; 1:500, Invitrogen) staining was performed overnight at 4 °C, and antibody binding specificity was revealed using a secondary antibody coupled to Alexa Fluor 594 (goat anti-rabbit Alexa Fluor 594, Invitrogen). Nuclear staining was performed by incubating cells with DAPI in PBS for 15 min. Images were acquired using a Zeiss confocal microscope. The morphological analysis of myotubes size was conducted using ImageJ software. The mean value of these measurements was used for the subsequent statistical analysis. The reported figures are representative of all the examined fields.

### Tissue processing and cell isolation for flow cytometric analysis

Muscle single-cell suspensions were obtained from mice, killed by cervical dislocation and perfused with PBS, and the hind limb musculature (tibialis anterior and gastrocnemius) was excised, weighed, cut into 1-mm pieces and digested with 1 mg/ml Collagenase type 4 (Worthington) for 1.5 h in a pre-warmed (37 °C) water bath with agitation. Muscles from each mouse were processed individually. The digested muscle preparation was first passed through a 70 μm cell strainer (BD Falcon) and then through a 40 μm cell strainer (BD Falcon), washed in Dulbecco’s modified eagle’s medium (DMEM, Life Technologies) 10% fetal bovine serum (FBS, Life Technologies) and resuspended in FACS buffer (PBS 2%FBS).

Splenocytes were obtained by grinding the spleen between the rough edges of 2 microscopy slides, while BM cells were obtained by centrifuging the hind limb bone inside a pierced 0.5 ml and 1.5 ml Eppendorf at 10.000 rpm for 30 sec. Single cells were counted with a hemocytometer and incubated with anti-CD16/32 (Biolegend, clone 93, 1 μl/test) to block non-specific binding to Fc receptors. Samples were then stained with the relevant antibodies including anti-CD45 (PE-Cy7, clone 30-F11,Biolegend); ant-CD11b (APC-Cy7, clone M1/70, Biolegend) anti-Ly6C (PerCP-Cy5.5, clone HK14, Biolegend); anti-Ly6g (PE, clone 1A8, eBioscience); anti-F4/80 (APC, clone BM8, Biolegend); anti-CD3 (FITC, clone 145-2C11, Biolegend) Samples were acquired with a CyAn ADP (DAKO), and acquired data were analysed using FlowJo software version 10.

### Statistical analysis

Data are presented as mean ± SEM. Comparisons were conducted using Student’s t test, assuming a two-tailed distribution, or two-way ANOVA multiple-comparison test, with significance defined as p < 0.05 (*), p < 0.01 (**), p < 0.001 (***) or p < 0.0001 (****).

## Results

### Dystrophic muscle injury alters the transcriptional response of mdx splenic monocytes to secondary stimulation

Inflammatory monocytes are the major immune cell type recruited to dystrophic muscle, where they differentiate into pro-inflammatory macrophages and contribute to chronic inflammation and progressive muscle degeneration (5, 27). We previously showed that the spleen is a major source of Ly6C^hi^ inflammatory monocytes in the *mdx* mouse model of DMD (3). To investigate whether chronic muscle injury induces transcriptional changes consistent with TI, we performed RNA-Seq on splenic monocyte-derived macrophages (splenic Mo-MP) from *mdx* and wild-type (WT) control mice, after *ex vivo* stimulation with lipopolysaccharide (LPS) or unstimulated control (Fig. 1A).

Principal Component Analysis (PCA) revealed a slight but clear separation between unstimulated WT and *mdx* Mo-MP (Fig. 1B) with the identification of 1610 differentially expressed genes (DEGs) (Fig. 1C-D). Interestingly, this segregation was drastically more pronounced upon LPS stimulation, resulting in the identification of 5024 DEGs in stimulated *mdx* versus WT Mo-MP (Fig. 1B-C). Gene ontology (GO) analysis performed on DEGs between stimulated *mdx* and WT Mo-MP showed the modulation of several biological processes involved in chromatin organization, RNA transcription and DNA damage response, indicating that Mo-MP derived from *mdx* mice exhibit a greater induction towards chromatin modifications and remodelling upon LPS stimulation (Fig. 1E).

To investigate the transcriptional differences underlying the activation programs of WT and *mdx* Mo-MP upon LPS stimulation, we compared stimulated and unstimulated WT and *mdx* Mo-MP and intersected the resulting sets of DEGs (Fig. 1F). Next, we performed GO analysis on the *mdx*-specific DEGs, with the goal of examining transcriptional differences related to TI. Interestingly, this analysis revealed a marked enrichment of pathways related to translation and ribosome biogenesis, accompanied by suppression of autophagy (Fig. 1G). These processes are typically associated with activation of mTOR-dependent anabolic programs, likely indicating a metabolic shift from a catabolic to a biosynthetically active state in *mdx* Mo-MP upon LPS stimulation.

To further investigate whether this transcriptional signature reflects a broader metabolic rewiring, we examined the expression of genes involved in major metabolic pathways. As shown in Fig. 1H, pathways known to be associated with TI, such as glycolysis, the TCA cycle, oxidative phosphorylation, purine metabolism, cholesterol biosynthesis, and amino acid metabolism (including tryptophan and glutamine), displayed a stronger deregulation in *mdx* compared to WT Mo-MP following LPS stimulation. Overall, these results suggest that dystrophic injury is associated with transcriptional changes in Mo-MP consistent with TI induction.

### Splenic Mo-MP from mdx mice exhibit trained immunity hallmarks

To verify these transcriptomic differences at the functional level, we quantified a set of inflammatory mediators by multiplex immunoassay on supernatants collected from cultured splenic Mo-MP following LPS stimulation.

Basal cytokine and chemokine levels were comparable between unstimulated *mdx* and WT Mo-MP; however, upon LPS stimulation, *mdx*-derived Mo-MP exhibited a significantly enhanced cytokine response compared to WT Mo-MP (Fig. 2A-B). The level of pro-inflammatory cytokines such as TNF-α and IL-1β was significantly greater in *mdx* compared with WT Mo-MP at 4 and 24 hours post-stimulation with LPS (Fig. 2A-B). Moreover, several chemokines were significantly modulated - either upregulated (i.e. MIP-1α, RANTES) or downregulated (i.e. KC) – in stimulated mdx Mo-MP, compared to stimulated WT (Fig. 2C-D). Consistent with these findings, macrophages differentiated from bone marrow (BM) monocytes of *mdx* mice at 4 weeks of age also showed a marked upregulation of multiple cytokines (Suppl. Figure 1A-B) and chemokines (Suppl. Figure 1C-D) compared to WT controls following LPS stimulation.

Altogether, these data indicate that chronic muscle injury in dystrophic mice is associated with a sustained, increased responsiveness of both splenic and BM monocytes upon secondary stimulation, consistent with TI.

### Inhibition of trained immunity enhances muscle regeneration.

Excessive activation of TI in the context of chronic inflammatory diseases, has been associated with worsening of the pathology (28, 29). Therefore, we sought to determine whether modulation of TI in the context of DMD may ameliorate the disease. To inhibit TI specifically in monocytes, we employed apolipoprotein A1 (apoA-I)–based, rapamycin-loaded nanobiologics (mTORi-NBs) (19).

We evaluated cellular uptake of mTORi-NB in our mouse model and confirmed that these nanobiologics are preferentially internalized by monocytes in the BM and the spleen (Suppl. Figure 2A-B).

We treated *mdx* mice starting at 4–6 weeks of age, during the peak necrotic and inflammatory phase of the disease, a time when monocytes isolated from the BM or spleen show a TI phenotype as shown above. mTORi-NBs were administered by intravenous injection twice weekly for two consecutive weeks, and animals were sacrificed at 6–8 weeks of age for subsequent analysis (Fig. 3A).

mTORi-NB therapy markedly reduced secretion of the major TI- associated cytokines TNF-α and IL-6 as determined by ELISA (Fig. 3B), and IL-1β expression (by RT- qPCR analysis) (Fig. 3C), compared with control (empty NBs). These results show that mTORi-NBs treatment reduces pro-inflammatory cytokine production and support the hypothesis of being effective at inhibiting TI in *mdx* Mo-MP. Of note, we could exclude that the observed reduction in pro-inflammatory cytokines production was due to cytotoxic effects of the treatment, as no significant differences in apoptotic cell death of isolated monocytes were observed between treated and control groups. Therefore, mTORi-NBs do not impair monocyte and Mo-MP viability (Suppl. Figure 2C-D).

To further examine the impact of this immune modulation on dystrophic muscle, we performed histological analysis of tibialis anterior (TA) muscles from *mdx* mice treated with mTORi-NB, empty nanobiologics (empty-NBs) or PBS. Morphometric analysis revealed a significantly higher proportion of smaller-diameter muscle fibers in mTORi-NB-treated mice (Fig. 3D-E) compared to control, while the overall muscle cross-sectional area (CSA) did not differ significantly between treatment groups (Suppl. Figure 3A). This shift in fiber size distribution indicates enhanced muscle regenerative activity following mTORi-NBs treatment.

Muscle regeneration was further evaluated by immunofluorescence staining for embryonic myosin heavy chain (eMHC), a marker of newly regenerating fibers. Consistent with the histological findings, mTORi-NB-treated mice exhibited a significant increase in the percentage of eMHC-positive fibers compared to the control groups (Fig. 3F-G). On the other hand, the overall extent of muscle necrosis, evaluated by IgG immunostaining, was not significantly affected by the treatment (Suppl. Figure 3B-C).

To further validate the pro-regenerative potential induced by the treatment, we FACS-sorted satellite cells (MuSCs), the muscle stem cells, from mTORi-NB, PBS, and empty-NB-treated *mdx* mice and examined their function *in vitro*. Although the total number of MuSCs did not differ among the experimental groups (Fig. 3H), myogenic differentiation markers *MyoD* and *MyoG* in MuSCs derived from mTORi-NB-treated mice were significantly upregulated, while expression levels of *Pax7*, a marker of MuSC identity and quiescence, were comparable across the experimental conditions (Fig. 3I). Upon *in vitro* differentiation, MuSCs isolated from mTORi-NB-treated mice displayed an enhanced capacity to form bigger myotubes with significantly higher number of nuclei compared to MuSCs derived from untreated mice (Fig. 3L-N). The fusion index (Fig. 3O), differentiation index (Fig. 3P) and total number of nuclei per field of view (Fig. 3Q) were also increased in the treatment group, although this increase did not reach statistical significance.

Collectively, these findings demonstrate that inhibition of TI using mTORi-NB treatment ameliorates dystrophic muscle pathology by enhancing muscle regeneration.

### Inhibition of trained immunity reduces fat accumulation in dystrophic muscle.

Given the progressive nature of DMD, improvement in muscle regeneration must be accompanied by effective control of pathological tissue remodelling, including fibrosis and fat infiltration. To investigate whether inhibition of TI could modulate these processes, we assessed fibrotic and adipogenic deposition in TA muscles of *mdx* mice.

While the treatment had no effect on the overall extent of fibrotic tissue deposition (Fig. 4A-B) it led to a significant reduction in lipid accumulation in TA muscles of *mdx* mice treated with mTORi-NBs compared with controls (Fig. 4C-D). This decrease in fat deposition suggests a shift toward a muscle microenvironment that is more permissive to regeneration and less prone to pathological remodelling.

To further investigate the cellular basis of these effects, we FACS-sorted fibro-adipogenic progenitors (FAPs), the cells responsible for fibrotic and adipose tissue deposition, from muscle of PBS-treated mice, empty-NB-treated mice, and mTORi-NB-treated *mdx* mice and analysed their differentiation potential *in vitro*.

Consistent with the reduced adipose accumulation observed in muscle sections *in vivo*, immunofluorescence analysis of differentiated FAPs revealed a significant decrease in perilipin expression in cells derived from mTORi-NB-treated mice (Fig. 4E-G).

Taken together, these findings indicate that mTORi-NB treatment selectively restrains adipogenic differentiation of FAPs without exacerbating fibrosis, thereby promoting a muscle microenvironment more conducive to regeneration rather than pathological remodelling.

## Inhibition of TI in Mo-MP directly impacts FAPs adipogenic potential

To evaluate whether inhibition of FAPs adipogenic potential upon mTORi-NB treatment was directly correlated with inhibition of TI in Mo-MP we assessed the capacity of trained *mdx* Mo-MP to influence FAPs fate *in vitro*. FAPs were isolated from WT mice and exposed, during both proliferation and differentiation phases, to conditioned medium (CM) derived from splenic Mo-MP isolated from *mdx* mice at different stages of the disease progression (2, 4, and 8 weeks of age), as well as from *mdx* mice treated with mTORi- NBs (Fig. 5A). CM was applied from the onset of culture (1:2 dilution with FAPs growth medium), and cells were allowed to differentiate for six days.

At the end of the differentiation period, adipogenic commitment was quantified by immunofluorescence analysis of perilipin-positive area (Fig. 5B). FAPs treated with CM from *mdx* Mo-MP displayed a progressive increase in perilipin expression that correlated with donor age and disease stage, indicating an enhanced pro-adipogenic influence of trained Mo-MP during disease progression. By contrast, FAPs exposed to CM derived from Mo-MP isolated from mTORi-NB-treated *mdx* mice exhibited a significant reduction in perilipin expression (Fig. 5C), indicating that TI inhibition in Mo-MP reduces adipogenic differentiation of FAPs.

## Discussion

Over the past decade trained immunity (TI) has emerged as a fundamental feature of innate immune cells, such as monocytes. While this process has been extensively characterized in the context of infections and vaccinations, where it is associated with improved outcomes, its role in chronic inflammatory conditions is less clear.

Here, we show that dystrophic muscle injury leads to transcriptional changes in monocytes at the level of the bone marrow and the spleen, with enrichment of pathways related to chromatin organization, transcriptional regulation, ribosome biogenesis, suppression of autophagy, and activation of mTOR-dependent anabolic programs. In addition, genes associated with multiple metabolic pathways were markedly upregulated and/or downregulated further supporting the presence of a broad metabolic rewiring in monocytes in the context of chronic muscle injury. Although epigenetic and metabolic alterations were not directly assessed in this study, the observed transcriptional landscape strongly supports the establishment of a primed functional state, which is a defining feature of TI (30, 31).

At a functional level, *mdx* monocytes showed markedly enhanced pro-inflammatory cytokine production upon secondary stimulation, a functional phenotype consistent with TI induction. These results are in line with the study by Bhattarai et al. (18) showing enhanced responsiveness of BM–derived macrophages in *mdx* mice. We extend these findings by demonstrating that not only BM, but also splenic monocytes are similarly reprogrammed, in line with our previous observation of the spleen as an important reservoir of inflammatory Ly6C^hi^ monocytes and a possible ‘training’ site during dystrophic muscle inflammation (3).

Consistent with the central role of chronic inflammation in DMD pathogenesis, glucocorticoids remain the current standard of care (32, 33). However, their broad immunosuppressive effects and long-term adverse outcomes underscore the need for more targeted immunomodulatory approaches (34). Here we show for the first time that inhibition of TI specifically in monocytes at the level of the BM and the spleen could represent a novel immunomodulatory approach in DMD. By selectively inhibiting mTOR signalling in monocytes, we were able to attenuate key features of TI and rebalance cytokine production by otherwise hyper-responsive *mdx* Mo-MP. Importantly, inhibition of TI resulted in improved regeneration and reduced fat accumulation within dystrophic muscles, highlighting the functional relevance of inflammatory monocytes reprogramming in driving DMD pathology. Macrophage derived cytokines are critically involved in the regulation of both SC regenerative potential and FAP responses to muscle damage (5). Cytokine hyperproduction by trained Mo-MP might therefore alter the otherwise finely tuned muscle microenvironment resulting in impaired regeneration, increased fibrosis and fat deposition. By rebalancing cytokine production by *mdx* Mo-MP through the inhibition of TI we were able to improve the regenerative capacity of satellite cells both *in vitro* and *in vivo*. Moreover, we show for the first time, that TI in *mdx* monocytes can directly influence FAPs differentiation fate through paracrine signaling. Soluble mediators released by *mdx* monocytes promoted adipogenic differentiation of FAPs in a disease stage-dependent manner, whereas inhibition of TI reversed this effect. These findings suggest that chronic activation of TI contributes to pathological FAPs differentiation, highlighting an altered crosstalk between Mo-MP and FAPs as a possible driver of fat deposition in dystrophic muscle.

Although we found no effect on muscle necrosis and fibrosis following a relatively short two-week treatment administered during the early stages of the disease, future studies are needed to assess the effect of a longer treatment duration as well as the effect on later stages of the disease.

## Conclusions

Dystrophic muscle injury is associated with the induction of TI in both BM and splenic monocytes characterised by hyperproduction of pro-inflammatory cytokines. Targeting TI specifically in monocytes through mTOR inhibition improves muscle regeneration and reduces fat accumulation. Overall, the results of this study suggest that inhibition of TI represents a promising novel therapeutic modality to slow DMD progression.

## Supplementary Material

This is a list of supplementary files associated with this preprint. Click to download.

- SupplementaryFigure1.pptx
- SupplementaryFigure2.pptx
- SupplementaryFigure3.pptx

## Figures and Tables

**Figure 1:**
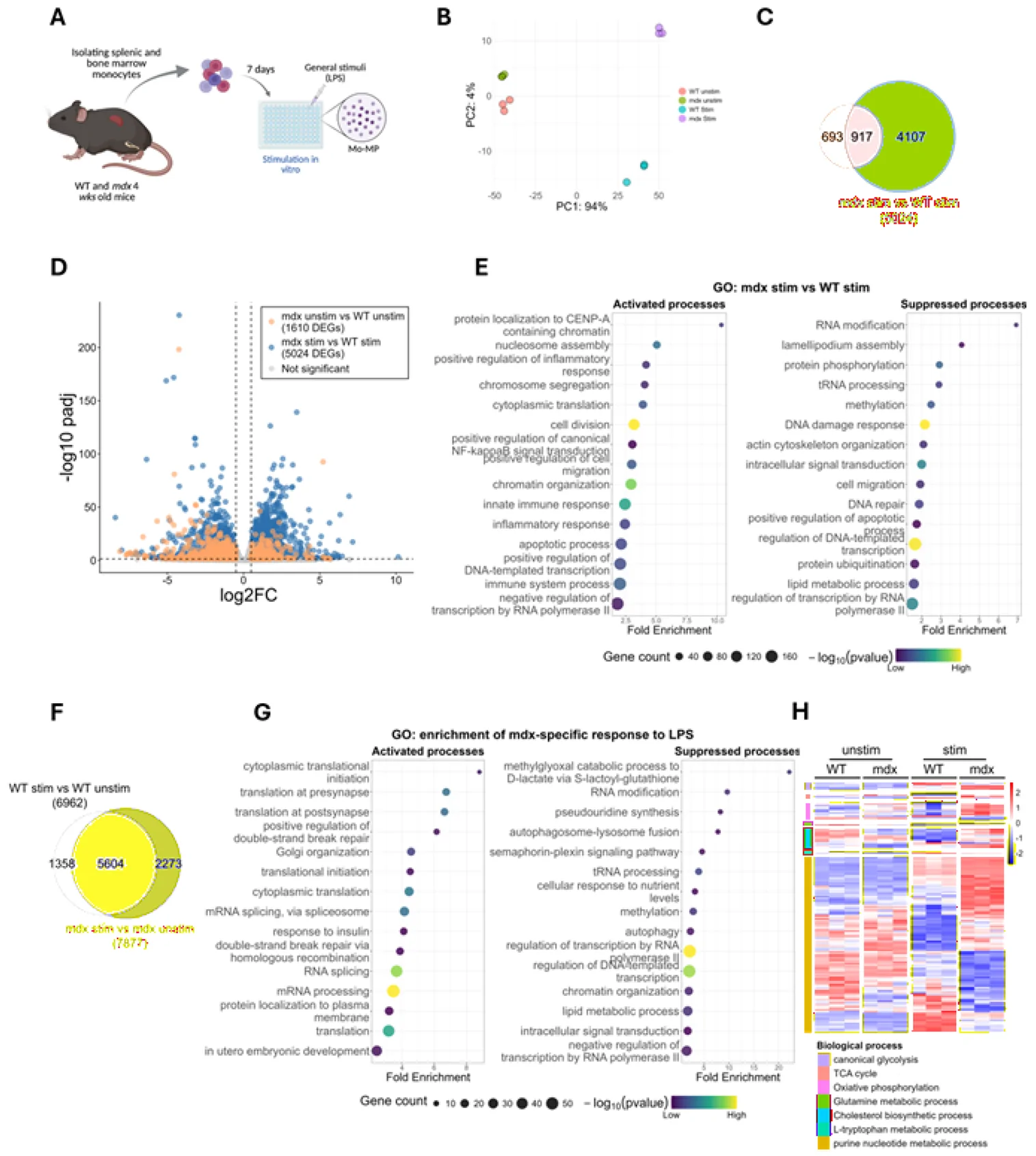
Splenic Mo-MP from *mdx* mice exhibit altered transcriptional response to secondary stimulation. **(A)** Schematic representation of experimental approach: Inflammatory Ly6C^hi^ monocytes were isolated from the spleen of *mdx* mice at 4 weeks of age (*mdx 4wks*) and age-matched wild-type mice (WT), differentiated *in vitro* for 7 days, and stimulated with LPS (100 ng/ml) for 24h, and gene expression analysed by RNA-Seq. **(B)** Principal Components Analysis of RNA-Seq performed on Mo-MP. **(C)** Venn diagram showing the intersection between DEGs in *mdx* unstimulated versus WT unstimulated Mo-MP and *mdx* stimulated versus WT stimulated Mo-MP. **(D)** Volcano plot showing differential gene expression (DEG). Significant DEGs in the *mdx* vs WT unstimulated comparison are highlighted in orange, while DEGs in the *mdx* vs WT stimulated comparison are shown in blue. Non-significant DEGs are shown in grey. **(E)** Gene ontology enrichment analysis (biological process) performed with DAVID on up- and down-regulated DEGs in *mdx* stimulated versus WT stimulated comparison. **(F)** Venn diagram showing the intersection between DEGs in *mdx* stimulated versus *mdx* unstimulated and WT stimulated versus WT unstimulated Mo-MP. **(G)** Gene ontology enrichment analysis (biological process) performed with DAVID on up- and down-regulated DEGs characterizing the *mdx* Mo-MP-specific response to LPS. **(H)** Heatmap showing the expression of genes involved in selected metabolic processes across the analyzed samples.

**Figure 2:**
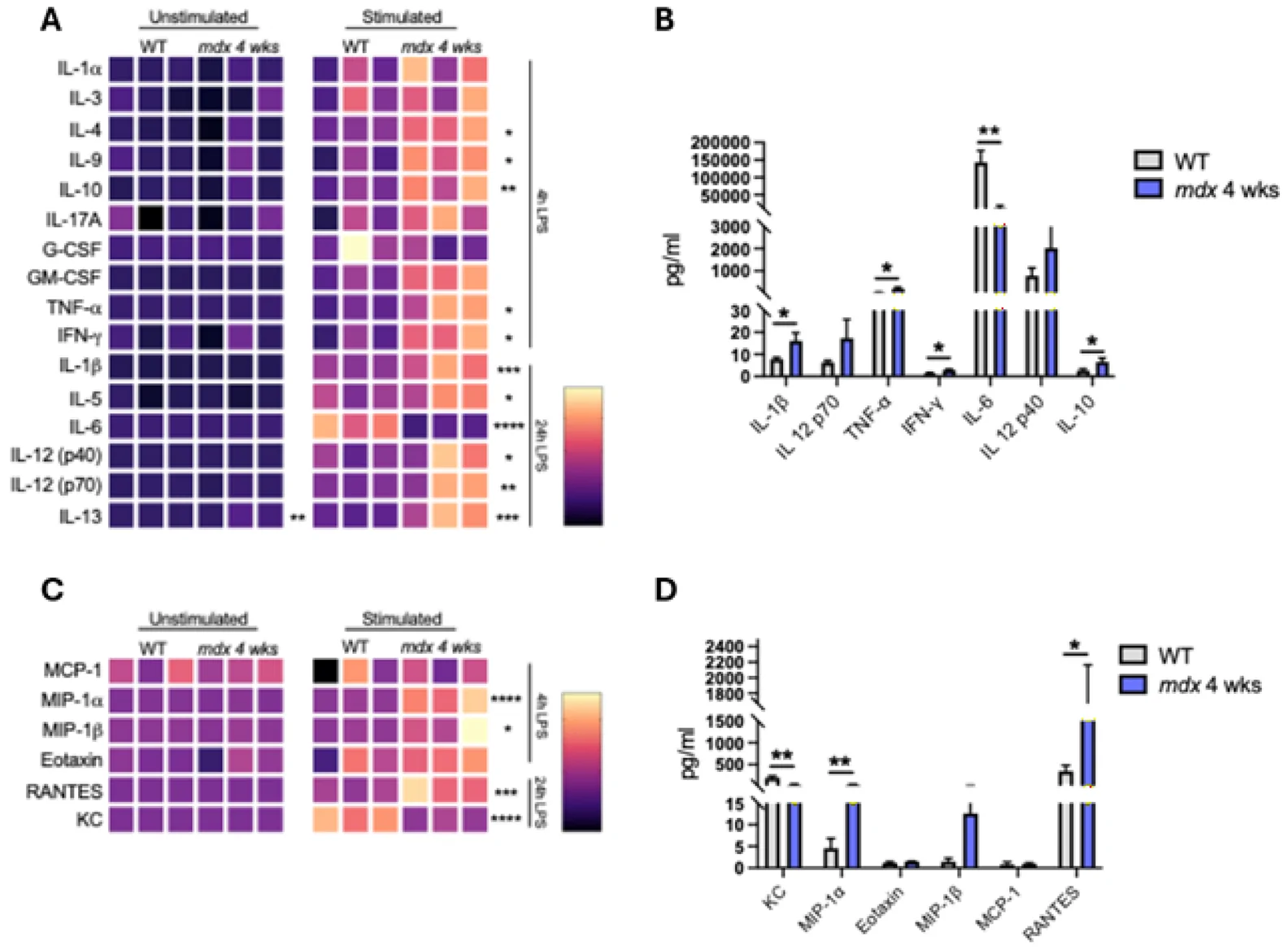
Splenic *mdx*Mo-MP exhibit trained immunity associated hyperproduction of cytokines and chemokines following secondary stimulation. **(A-B)** Multiplex ELISA analysis shows no differences in the levels of cytokines in the supernatant of splenic unstimulated *mdx 4 wks* Mo-MP compared to WT control but increased levels of cytokines in the supernatant of splenic Mo-MP of *mdx*mice at 4 weeks of age compared to WT control, after stimulation with LPS (WT n=3; *mdx 4 wks* n=3). **(C-D)** Multiplex ELISA analysis shows no differences in the levels of chemokines in the supernatant of unstimulated *mdx 4 wks* Mo-MP compared to WT control but increased levels of chemokines in the supernatant of splenic Mo-MP of *mdx* mice at 4 weeks of age compared to WT control, after stimulation with LPS (100 ng/ml) (WT n=3;*mdx 4 wks*n=3). Data are presented as mean ± SEM *p < 0.05; ** p< 0.01; *** p< 0.001; **** p < 0.0001. Two-way ANOVA was used for multiple comparison, and unpaired two- tailed t test was applied for pairwise analyses.

**Figure 3:**
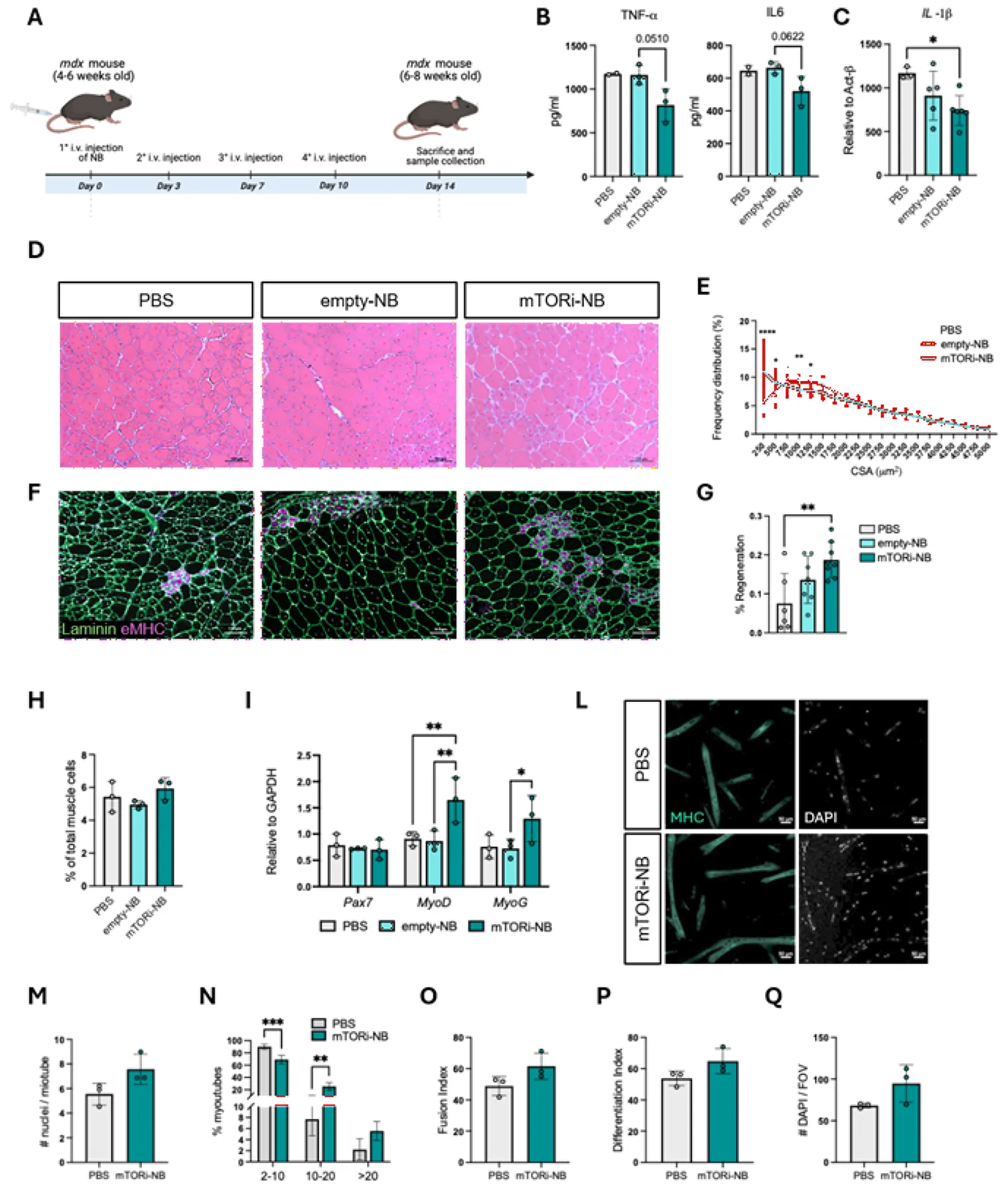
Inhibition of trained immunity enhances muscle regeneration. **(A)** Experimental design: *mdx*mice were treated with apolipoprotein A based, rapamycin-loaded nanobiologics (mTORi-NBs), unloaded (empty-NBs) or PBS by twice weekly i.v. injections for two weeks starting at 4-6 weeks of age. **(B)** TNF-aand IL-6 levels in the supernatant of BM Mo-MP from treated *mdx* mice following LPS stimulation for 4h (PBS n=2;empty NBs n=3; mTORi-NB n=3) **(C)** RT- qPCR analysis IL-1b on expression levels in BM Mo-MP from treated *mdx* mice following LPS stimulation for 4h. (PBS n=3; empty NBs n=5; mTORi-NB n=6) **(D)** H&E staining on TA muscle of *mdx*mice treated with PBS, empty-NB and mTORi-NB. **(E)** Frequency distribution of muscle fibers in TA muscle of *mdx* PBS, empty-NB and mTORi-NB- treated mice (PBS n=6; empty-NB n=7; mTORi-NB n=9). **(F)** Representative immunostaining and **(G)** Quantification of eMHC+ fibers (purple) and laminin (green) of PBS control, empty-NB and mTORi-NB- treated mice (PBS n=6; empty-NB n=7; mTORi-NB n=8). **(H)** Percentage of MuSCs relative to total muscle cells (PBS n=3; empty-NB n=3; mTORi-NB n=3). **(I)** RT- qPCR analysis of *Pax7, MyoD* and *MyoG* expression levels in satellite cells at day 0 isolated from PBS control, empty-NB and mTORi-NB- treated mice (PBS n=3; empty-NB n=3; mTORi-NB n=3). **(L)** Representative immunostaining of MHC+ (green) and DAPI (white) myotubes from PBS control and mTORi-NB treated mice. **(M)** Quantification of nuclei per myotube. **(N)**Quantification of the percentage of myotubes with 2 to 10, 10 to 20, or more than 20 nuclei. **(O)** Quantification of Fusion Index [(number of nuclei within myotubes/ total number of nuclei) *100]. **(P)** Quantification of Differentiation Index [(number of MHC+ nuclei/ total number of nuclei) *100]. **(Q)** Quantification of number of nuclei per field of view (PBS n=3; empty-NB n=3; mTORi-NB n=3). Data are presented as mean ± SEM *p<0.05; **p<0.01; ***p<0.001 by one-way and two-way ANOVA test.

**Figure 4:**
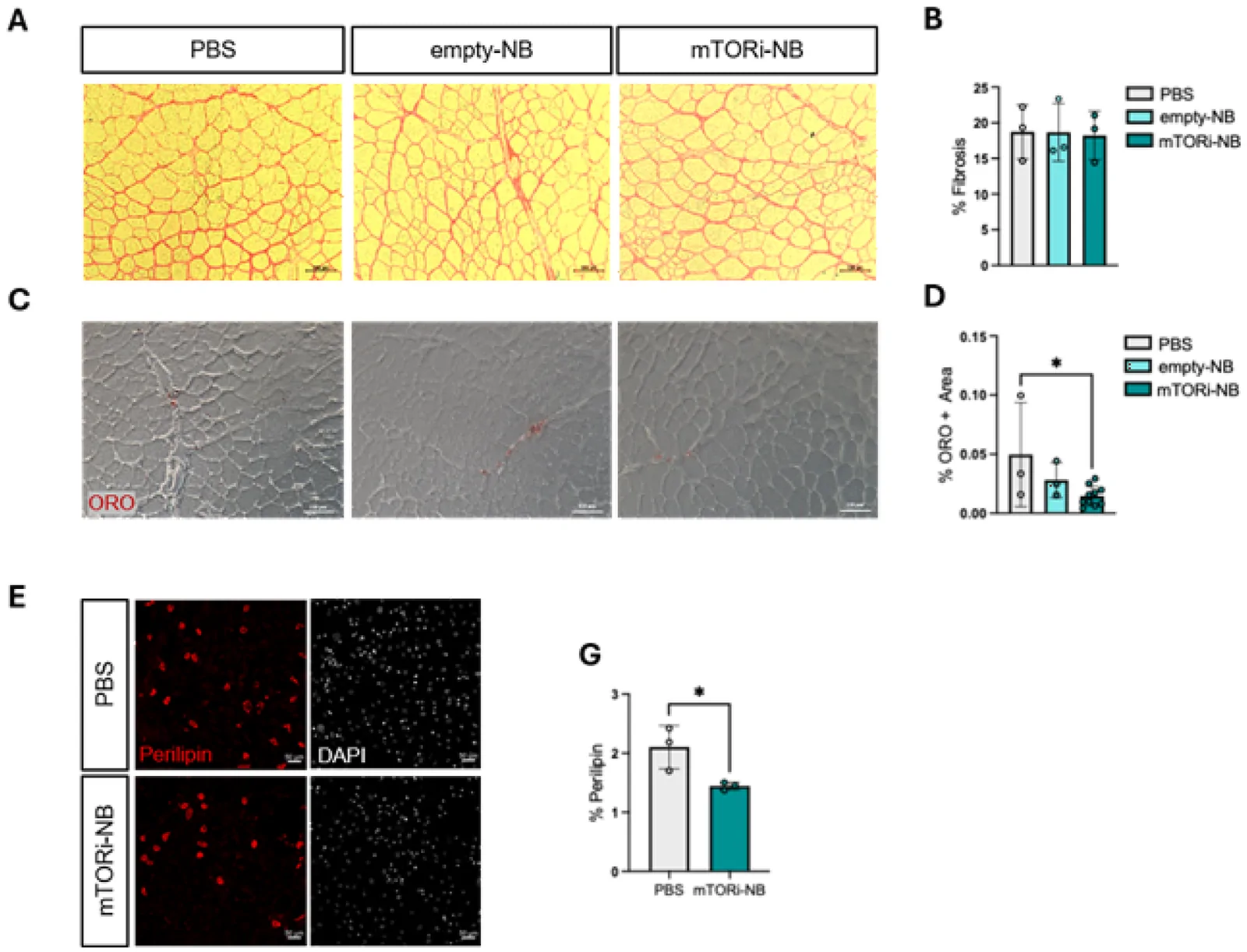
mTORi-NB treatment reduces fatty tissue deposition. **(A)** Representative images and **(B)** Quantification of sirius-red staining of PBS control, empty-NB and mTORi-NB- treated mice. (PBS n=3; empty-NB n=3; mTORi-NB n=3) **(C)** Representative immunostaining and **(D)** Quantification of Oil Red O (ORO) staining (red) of PBS control, empty-NB and mTORi-NB-treated mice (PBS n=3; empty-NB n=3; mTORi-NB n=10). **(E)** Representative immunostaining and **(G)** Quantification of Perilipin (red) and DAPI (white) in differentiated FAPs isolated from PBS control and mTORi-NB treated mice. Data are presented as mean ± SEM *p<0.05; **p<0.01; ***p<0.001; ****p<0.0001 by one-way, two-way ANOVA and student t test.

**Figure 5:**
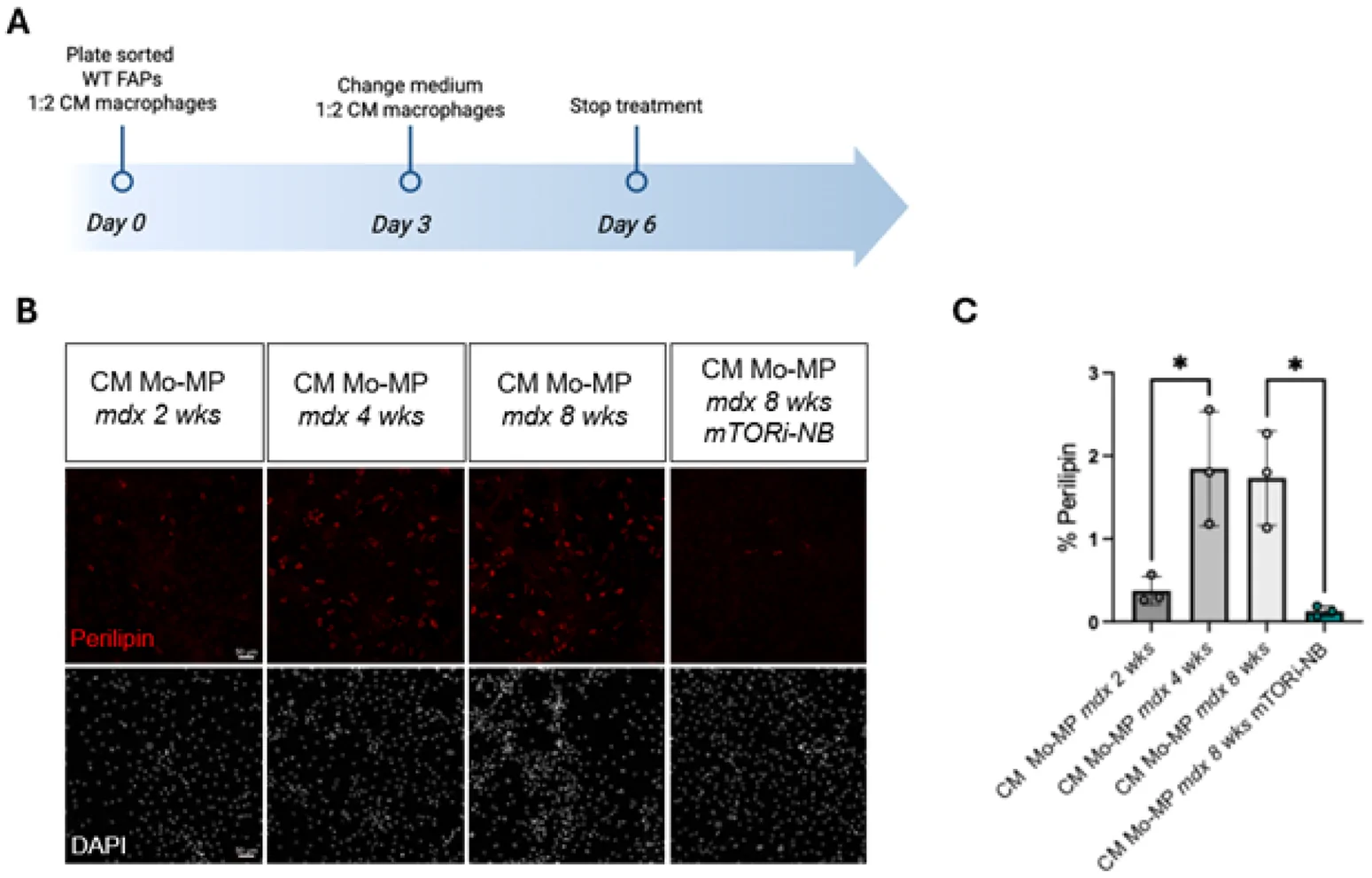
Inhibition of TI in Mo-MP reduces FAPs adipogenic potential **(A)** FAPs were isolated from wild-type mice, cultured in growth medium 1:2 with CM of *mdx* Mo-MP at different stages of the disease (2,4,8 weeks of age) and *mdx* treated with mTORi-NBs, for 6 days. **(B)** Representative immunostaining of perilipin positive cells (red) and DAPI (white) of FAPs treated with CM from from Mo-MP from *mdx* mice at different stages of the disease compared to treated control. **(C)** Quantification of percentage of perilipin positive area of FAPs treated with CM from MO-MP from *mdx* mice at different stages of the disease compared to treated control. Data are presented as mean ± SEM *p<0.05 by One way ANOVA test.

## Abbreviations

TI: Trained immunity
DMD: Duchenne muscular dystrophy
DAMPs: Damage-associated molecular patterns
BM: Bone marrow
LPS: Lipopolysaccharide
mTOR: Mammalian target of rapamycin
mTORi-NB: Mammalian target of rapamycin inhibitor- nanobiologics
FAPs: Fibro-adipogenic progenitors
MuSC: Muscle stem-cells
Mo-MP: Monocyte-derived macrophages
TNF-a: Tumor necrosis factor a
IL: Interleukin
G-CSF: Granulocyte colony stimulating factor
GM-CSF: Granulocyte monocyte colony stimulating factor
IFN: Interferon
MCP-1: Monocyte chemoattractant protein-1
MIP: Macrophage inflammatory protein
KC: Keratinocyte-derived chemochine
M-CSF: Monocyte colony stimulating factor
CSA: Cross-sectional area
DEGs: Differentially expressed genes
PCA: Principal component analysis
GO: Gene ontology
ApoA1: Apolipoprotein A1
eMHC: embryonic myosin heavy chain

## References

[1] Mendell JR, Shilling C, Leslie ND, Flanigan KM, Al-Dahhak R, Gastier-Foster J, Evidence-based path to newborn screening for duchenne muscular dystrophy. Ann Neurol. 2012 Mar;71(3):304–13. doi:10.1002/ana.23528. 22451200

[2] Rosenberg MI, Georges SA, Asawachaicharn A, Analau E, Tapscott SJ. MyoD inhibits Fstl1 and Utrn expression by inducing transcription of miR-206. Journal of Cell Biology. 2006 Oct 9;175(1):77–85. doi:10.1083/jcb.200603039. PMC2064500 17030984

[3] Rizzo G, Di Maggio R, Benedetti A, Morroni J, Bouche M, Lozanoska-Ochser B. Splenic Ly6Chi monocytes are critical players in dystrophic muscle injury and repair. JCI Insight. 2020 Jan 30;5(2). doi: 10.1172/jci.insight.130807. PMC7098719 31874104

[4] Martinez CO, Mchale MJ, Wells JT, Ochoa O, Michalek JE, Mcmanus LM, Regulation of skeletal muscle regeneration by CCR2-activating chemokines is directly related to macrophage recruitment. Am J Physiol Regul Integr Comp Physiol. 2010;299:832–42. doi:10.1152/ajpregu.00797.2009.-Muscle PMC2944434 20631294

[5] Tidball JG. Regulation of muscle growth and regeneration by the immune system. Nature Reviews Immunology. Nature Publishing Group; 2017. p. 165–78. doi:10.1038/nri.2016.150. PMC5452982 28163303

[6] Arnold L, Henry A, Poron F, Baba-Amer Y, Van Rooijen N, Plonquet A, Inflammatory monocytes recruited after skeletal muscle injury switch into antiinflammatory macrophages to support myogenesis. Journal of Experimental Medicine. 2007 May;204(5):1057–69. doi:10.1084/jem.20070075. PMC2118577 17485518

[7] Lemos DR, Babaeijandaghi F, Low M, Chang CK, Lee ST, Fiore D, Nilotinib reduces muscle fibrosis in chronic muscle injury by promoting TNF-mediated apoptosis of fibro/adipogenic progenitors. Nat Med. 2015 Jul 9;21(7):786–94. doi:10.1038/nm.3869. 26053624

[8] Netea MG. Training innate immunity: The changing concept of immunological memory in innate host defence. European Journal of Clinical Investigation. 2013. p. 881–4. doi:10.1111/eci.12132 23869409

[9] Quintin J, Saeed S, Martens JHA, Giamarellos-Bourboulis EJ, Ifrim DC, Logie C, Candida albicans infection affords protection against reinfection via functional reprogramming of monocytes. Cell Host Microbe. 2012 Aug 16;12(2):223–32. doi: 10.1016/j.chom.2012.06.006. PMC3864037 22901542

[10] Vuscan P, Kischkel B, Joosten LAB, Netea MG. Trained immunity: General and emerging concepts. Immunological Reviews. John Wiley and Sons Inc; 2024. p. 164–85. doi:10.1111/imr.13326. 38551324

[11] Hajishengallis G, Netea MG, Chavakis T. Trained immunity in chronic inflammatory diseases and cancer. Nature Reviews Immunology. Nature Research; 2025. p. 497–514. doi:10.1038/s41577-025-01132-x. PMC12450111 39891000

[12] Goodridge HS, Ahmed SS, Curtis N, Kollmann TR, Levy O, Netea MG, Harnessing the beneficial heterologous effects of vaccination. Nature Reviews Immunology. Nature Publishing Group; 2016. p. 392–400. doi:10.1038/nri.2016.43. PMC4931283 27157064

[13] Moorlag SJCFM, Khan N, Novakovic B, Kaufmann E, Jansen T, van Crevel R, β-Glucan Induces Protective Trained Immunity against Mycobacterium tuberculosis Infection: A Key Role for IL-1. Cell Rep. 2020 May 19;31(7). doi:10.1016/j.celrep.2020.107634. PMC7242907 32433977

[14] Christ A, Günther P, Lauterbach MAR, Duewell P, Biswas D, Pelka K, Western Diet Triggers NLRP3-Dependent Innate Immune Reprogramming. Cell. 2018 Jan 11;172(1–2):162–175.e14. doi:10.1016/j.cell.2017.12.013. PMC6324559 29328911

[15] Van Der Heijden CDCC, Noz MP, Joosten LAB, Netea MG, Riksen NP, Keating ST. Epigenetics and Trained Immunity. Antioxidants and Redox Signaling. Mary Ann Liebert Inc.; 2018. p. 1023–40. doi:10.1089/ars.2017.7310. PMC6121175 28978221

[16] Bekkering Siroon, Arts Rob J.W., Novakovic Boris…, Stunnenberg Jenk, Riksen Niels P., Netea Mihai G.. Metabolic Induction of Trained Immunity through the Mevalonate Pathway. 2018. 10.1016/j.cell.2017.11.025 29328908

[17] Bekkering S, Domínguez-Andrés J, Joosten LAB, Riksen NP, Netea MG. Trained Immunity: Reprogramming Innate Immunity in Health and Disease. 2021. Annu Rev Immunol. 2021 Apr 26;39:667–693. doi: 10.1146/annurev-immunol-102119-073855. 33637018

[18] Bhattarai S, Li Q, Ding J, Liang F, Gusev E, Lapohos O, TLR4 is a regulator of trained immunity in a murine model of Duchenne muscular dystrophy. Nat Commun. 2022 Dec 1;13(1). doi:10.1038/s41467-022-28531-1. PMC8847425 35169163

[19] Teunissen AJP, Van Leent MMT, Prévot G, Brechbühl EES, Pérez-Medina C, Duivenvoorden R, Targeting Trained Innate Immunity with Nanobiologics to Treat Cardiovascular Disease. Arterioscler Thromb Vasc Biol. 2021 Jun 1;41(6):1839–50. doi:10.1161/ATVBAHA.120.315448. PMC8159873 33882685

[20] Priem B, van Leent MMT, Teunissen AJP, Sofias AM, Mourits VP, Willemsen L, Trained Immunity-Promoting Nanobiologic Therapy Suppresses Tumor Growth and Potentiates Checkpoint Inhibition. Cell. 2020 Oct 29;183(3):786–801.e19. doi:10.1016/j.cell.2020.09.059. PMC8074872 33125893

[21] Van Leent MMT, Meerwaldt AE, Berchouchi A, Toner YC, Burnett ME, Klein ED, A modular approach toward producing nanotherapeutics targeting the innate immune system. Sci. Adv. 2021. doi: 10.1126/sciadv.abe7853 PMC7935355 33674313

[22] Ochando J, Mulder WJM, Madsen JC, Netea MG, Duivenvoorden R. Trained immunity — basic concepts and contributions to immunopathology. Nature Reviews Nephrology. Nature Research; 2023. p. 23–37. doi:10.1038/s41581-022-00633-5. PMC9575643 36253509

[23] Braza MS, van Leent MMT, Lameijer M, Sanchez-Gaytan BL, Arts RJW, Pérez-Medina C, Inhibiting Inflammation with Myeloid Cell-Specific Nanobiologics Promotes Organ Transplant Acceptance. Immunity. 2018 Nov 20;49(5):819–828.e6. doi:10.1016/j.immuni.2018.09.008. PMC6251711 30413362

[24] van Leent MMT, Beldman TJ, Toner YC, Lameijer MA, Rother N, Bekkering S, Prosaposin mediates inflammation in atherosclerosis. Sci Transl Med. 2021 Mar 10;13(584). doi:10.1126/scitranslmed.abe1433. PMC8209679 33692130

[25] Domínguez-Andrés J, Arts RJW, Bekkering S, Bahrar H, Blok BA, de Bree LCJ, In vitro induction of trained immunity in adherent human monocytes. STAR Protoc. 2021 Mar 19;2(1). doi:10.1016/j.xpro.2021.100365. PMC7921712 33718890

[26] Morroni J, Benedetti A, Esposito L, De Bardi M, Borsellino G, Riera CS, Injury-experienced satellite cells retain long-term enhanced regenerative capacity. Stem Cell Res Ther. 2023 Dec 1;14(1). doi:10.1186/s13287-023-03492-4. PMC10496398 37697344

[27] Villalta SA, Rinaldi C, Deng B, Liu G, Fedor B, Tidball JG. Interleukin-10 reduces the pathology of mdx muscular dystrophy by deactivating M1 macrophages and modulating macrophage phenotype. Hum Mol Genet. 2011 Feb;20(4):790–805. doi:10.1093/hmg/ddq523. PMC3024048 21118895

[28] Zhong C, Yang X, Feng Y, Yu J. Trained Immunity: An Underlying Driver of Inflammatory Atherosclerosis. Frontiers in Immunology. Frontiers Media S.A.; 2020. doi:10.3389/fimmu.2020.00284. PMC7046758 32153588

[29] Bekkering S, Saner C, Riksen NP, Netea MG, Sabin MA, Saffery R, Trained Immunity: Linking Obesity and Cardiovascular Disease across the Life-Course? Trends in Endocrinology and Metabolism. Elsevier Inc.; 2020. p. 378–89. doi:10.1016/j.tem.2020.01.008. 32305098

[30] Tercan H, Riksen NP, Joosten LAB, Netea MG, Bekkering S. Trained Immunity ; Long-term adaptation in innate immune responses. Arterioscler Thromb Vasc Biol. 2021 Jan 1;41(1):55–61. doi:10.1161/ATVBAHA.120.314212. 33086868

[31] Saeed S, Quintin J, Kerstens HHD, Rao NA, Aghajanirefah A, Matarese F, Epigenetic programming of monocyte-to-macrophage differentiation and trained innate immunity. Science (1979). 2014 Sep 26;345(6204). doi:10.1126/science.1251086. PMC4242194 25258085

[32] Waldrop MA, Flanigan KM. Update in Duchenne and Becker muscular dystrophy. Current Opinion in Neurology. Lippincott Williams and Wilkins; 2019. p. 722–7. doi:10.1097/WCO.0000000000000739. 31343429

[33] Matthews E, Brassington R, Kuntzer T, Jichi F, Manzur AY. Corticosteroids for the treatment of Duchenne muscular dystrophy. Cochrane Database of Systematic Reviews. John Wiley and Sons Ltd; 2016. doi:10.1002/14651858.CD003725.pub4. PMC8580515 27149418

[34] Hussein MR, Hamed SA, Mostafa MG, Abu-Dief EE, Kamel NF, Kandil MR. The effects of glucocorticoid therapy on the inflammatory and Dendritic cells in muscular dystrophies. Int J Exp Pathol. 2006 Dec;87(6):451–61. doi:10.1111/j.1365-2613.2006.00470.x. PMC2517389 17222213

